# Expression analysis data of T-cell surface antigen CD2 gene from rock bream (*Oplegnathus fasciatus*)

**DOI:** 10.1016/j.dib.2019.103832

**Published:** 2019-03-26

**Authors:** Dong-Hee Cho, Mu-Chan Kim, Chan-Il Park

**Affiliations:** Institute of Marine Industry, College of Marine Science, Gyeongsang National University, 455, Tongyeong 650-160, Republic of Korea

## Abstract

This data article reports the expression level of T-cell surface antigen CD2 gene in organs from normal rock bream through quantitative real-time PCR. We also report the expression level of CD2 gene when anthropogenic infection with bacterial or viral pathogens was induced. The expression pattern of CD2 gene in normal rock bream was proved to be highly expressed in hematopoietic cells involved in the production and development of peripheral blood leukocytes (PBLs) and immune cells. It also proved that it maintains high expression for normal immunity in gills, skin, and intestines exposed directly to the environment. The expression pattern of CD2 gene in pathogenic infection has proven that CD2 is a factor involved in immunity. These data are considered to be a basic study of teleost immune system and will contribute to the study of fish blood cells.

**Table:** Specifications table

Subject area	Biology
More specific subject area	Gene expression analysis
Type of data	Figure
How data was acquired	Real-time PCR (Thermal Cycler DICE Real-Time System (Takara Bio Inc.) using SYBR™ Green Master Mix (Takara, Kyoto, Japan))
Data format	Analyzed
Experimental factors	Samples were obtained from healthy fish and *E. tarda* (1.5 × 10^5^ cells/fish), *S. iniae* (1.5 × 10^5^ cells/fish), and RSIV (1.1 × 10^4^ copies/fish) injected intraperitoneally in fish.
Experimental features	Changes in the Expression of CD2 Gene for Pathogen Infection.
Data source location	Gyeongsang National University, Tongyeong, Republic of Korea
Data accessibility Related research article	Data is within this article. D. H. Cho, J. S. Bae a, J. M. Jeong, H. J. Han, D. C. Lee, M. Y. Cho, S. H. Jung, D. H. Kim, C. I. Park, The first report of CD2 associated protein gene, in a teleost (Rock bream, *Oplegnathus fasciatus*): An investigation of the immune response upon infection with several pathogens., Fish and Shellfish Immunology 67 (2017) 1–6 [1].

**Table:** 

**Value of the data** • This is the first report on CD2, the membrane surface protein of T and NK cells in rock bream. • CD2 is expressed on the surface of almost all T cells and natural killer (NK) cells in mammals and the interaction with the ligand provides an optimal interval for direct antigen challenge or antigen-presenting cells and T cell contact. • The CD2 primers and results provided in this data will help to understand the immune signaling system of fish T and NK cells to be studied in the future. • Expression analysis results in rock bream can be further used for comparative analysis with expression assays in other fish.

## 1 Data

**Fig. 1:**
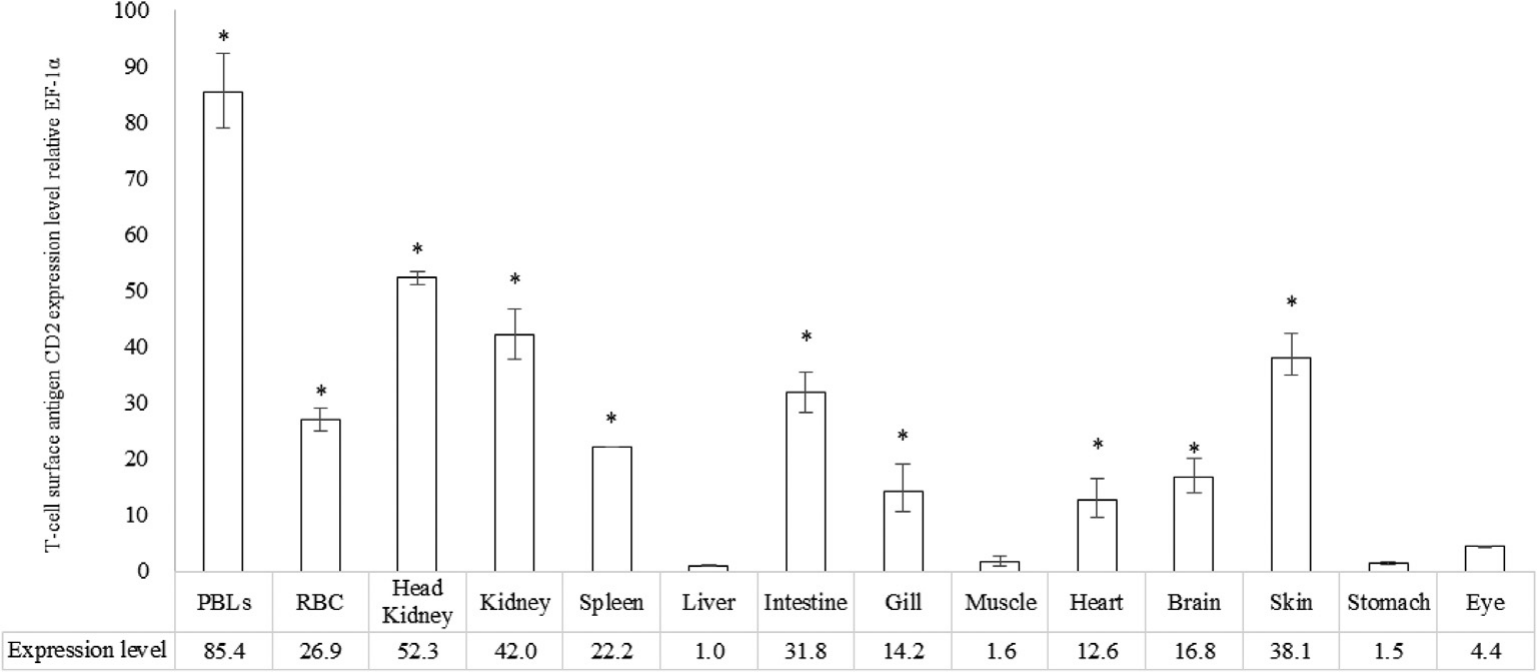
Detection of CD2 genes in different tissues of healthy rock bream by real-time PCR. EF-1α was used for normalizing the real-time PCR results. Data are presented as the mean ± SD from three independent cDNA samples with three replicates from each sample. Asterisks (*) indicate significant differences (p < 0.05) compared to liver.

CD2 mRNA expression levels in various tissues of normal rock bream bodies were checked. On the basis of the liver with the lowest expression level, PBLs showed the highest expression level. In addition, high gene expression levels were identified in the head kidney, kidney, skin and intestines (Fig. 1).

**Fig. 2:**
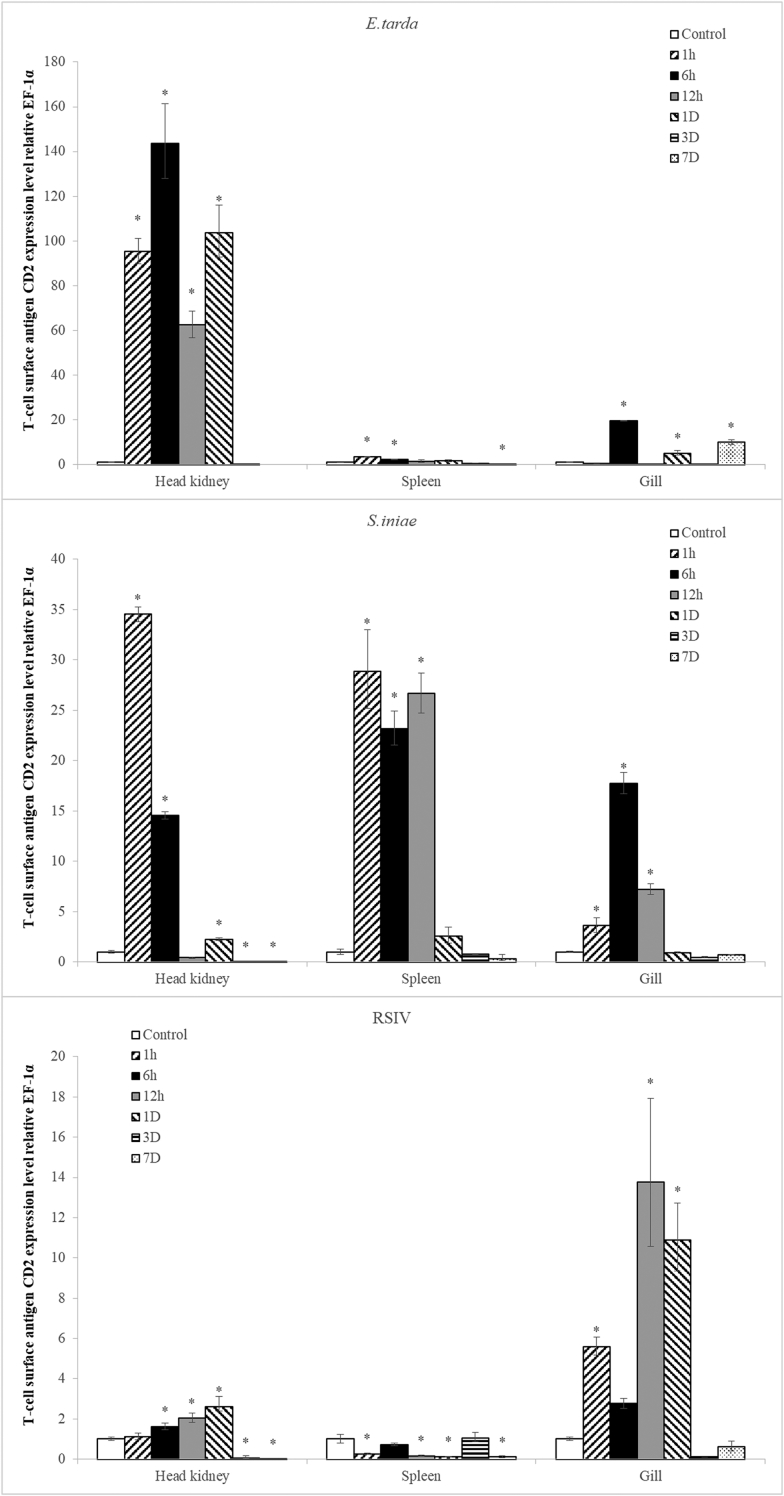
Gene expression of CD2 in the head kidney, spleen and gill after infection with *E. tarda*, *S. iniae*, and RSIV. Levels of CD2 transcripts were quantified relative to that of EF-1α levels. Data are presented as the mean ± SD from three independent cDNA samples with three replicates for each sample. Asterisks (*) represent significant differences compared to the control (0h) group by ANOVA (p < 0.05).

After infecting rock bream with bacteria and viruses, the expression patterns of CD2 gene in the head kidney, spleen, and gill were checked with quantitative real-time PCR. In the head kidney, CD2 increased 1 hour after infection with *Edwardsiella tarda*, maintained the same value until day 1, and decreased to the normal level 3 days later. In the case of infection with *Streptococcus iniae*, CD2 increased in the head kidney and spleen, respectively, 1 hour after the infection. It increased in the gill 6 hours after the infection and decreased to the normal levels compared to those of the control group one day later. After the infection with RSIV, the gill showed similar results to those of the kidney in the *E. tarda* experimental group, but the expression level was not high (Fig. 2).

## 2 Experimental design, materials, and methods

We sampled aseptically peripheral blood leukocytes (PBLs), red blood cells (RBCs), head kidney, kidney, spleen, liver, intestine, gill, muscle, heart, brain, skin, stomach and eye from three healthy rock bream [2]. PBLs and RBCs were separated from 5 ml of 51% Percoll solution (Percoll: 10 × Phosphate buffer saline (PBS): 1 × PBS at a ratio of 51: 9: 40) (Sigma, USA) and 5 ml of RPMI was dispensed into the layers, and centrifuged at 500 g for 20 minutes. The separated PBLs and RBCs were washed three times with 1 × PBS, and the pellet obtained by centrifugation was used for the experiment [3].

After the addition of 500 μl of TRIzol, the mixture was homogenized and vortexed with 100 μl of chloroform (Invitrogen, Carlsbad, CA, USA) and centrifuged at 14,000 rpm for 10 minutes. The supernatant was transferred to a new e-tube and equilibrated with PCI (Phenol: Chloroform: Isoamylalcohol) and centrifuged at 14,000 rpm for 10 min. After transferring the supernatant to a new e-tube, add 500 μl of isopropanol (Sigma, USA), 3 μl of Dr. Gen (TaKaRa, Japan) and 30 μl of 3 M sodium acetate (TaKaRa, Japan) Respectively. After removing all supernatant, 600 μl of 75% DEPC ethyl alcohol was added and centrifuged at 14,000 rpm for 5 minutes. Finally, the supernatant was removed and dried naturally at room temperature for 10–15 minutes, followed by addition of DEPC DDW (30–40 μl). The isolated total RNA was treated with DNase according to manufacturer's instructions using RNase-free DNase (Promega, USA). 1 μl of total RNA, 1 μl of anchored-oligo (dT) primer and 11 μl of water The mixture was reacted at 65 °C for 10 minutes and then on ice for 5 minutes. cDNA was synthesized by mixing 4 μl of Transcriptor Reverse Transcriptase Reaction Buffer, 0.5 μl of Protector RNase Inhibitor, 2 μl of Deoxynucleotide Mix and 0.5 μl of Transcriptor Reverse Transcriptase for 30 min at 55 °C and 85 °C for 5 min [1].

Specific primer sets used in quantitative real-time PCR were Primer3 ver. 3 based on cDNA full-length sequences 0.4.0 (http://bioinfo.ut.ee/primer3-0.4.0/) (forward: 5′- ACTGTTGTAGCAGCGGTAGC -3′, reverse: 5′- AGCTCCCCTTTATCCCTGAG -3′).

For quantitative real-time PCR, 1 μl of cDNA template, 1 μl of forward and reverse primers, 12.5 μl of SYBR Green, and 9.5 μl of DDW were mixed in a total volume of 25 μl using SYBR Green Master Mix (TaKaRa, Shiga, Japan) according to the manufacturer and incubated at 50 °C for 4 minutes After initial denaturation at 95 °C for 10 min, reaction was carried out 45 times at 95 °C for 20 sec and 60 °C for 1 min. Finally, 95 °C for 15 sec, 60 °C for 30 sec, 95 And dissociated at 15 °C for 15 seconds. The degree of CD2 mRNA expression was compared with the expression level of elongation factor (EF) -1α (forward: 5′- CCCCTGCAGGACGTCTACAA -3′, reverse: 5′- AACACGACCGACGGGTACA -3′) mRNA and three repetitions were performed for each gene for the accuracy of the experiment. The expression level of each gene was calculated by the 2^–ΔΔCT^ method, and all data were expressed as mean ± SD. Significant differences between the groups were confirmed by one-way analysis of variance (ANOVA) test (p < 0.05).

The gene expressions of CD2 in head kidney and spleen of infected rock bream were measured by qRT-PCR, according to our previous study with minor modification. Briefly, fish in the experimental group were injected intraperitoneally with *Edwardsiella tarda* FP4130 (*E. tarda*) (1.2 × 10^5^ cells/fish), *Streptococcus iniae* FP5228 (*S. iniae*) (1.3 × 10^5^ cells/fish), and RSIV (1.5 × 10^4^ copies/fish). Control fish were injected with the same volume of phosphate-buffered saline (PBS). Three specimens were randomly selected from 1 h, 6 h, 12 h, 1 D, 3 D and 7 D after injection of pathogens and tissues of kidney, spleen and gill were extracted. Total RNA isolation, cDNA synthesis, and quantitative real-time PCR were performed as described for tissue expression in normal recipients.
